# TruckSim-based study of the rollover accident mechanisms of container semitrailers on freeway interchange loop ramps

**DOI:** 10.1371/journal.pone.0309139

**Published:** 2024-09-04

**Authors:** Shibo Zhang, Xuefeng Yang, Yishan Liu, Li Wang, Pingfei Li, Hui Li, Yong Luo, Yi Li, Qiaoyuan Liu

**Affiliations:** 1 School of Automobile and Transportation, Xihua University, Chengdu, China; 2 Sichuan Xihua Jiaotong Forensic Center, Chengdu, China; 3 Sichuan Vocational and Technical College of Communications, Chengdu, China; 4 Traffic Police Headquarters of Sichuan Public Security Department, Chengdu, China; Southwest Jiaotong University, CHINA

## Abstract

In this study, to investigate the factors affecting container semitrailer rollovers while driving on interchange loop ramps, a simulation model was established in TruckSim on the basis of data collected from an in-depth investigation of the vehicle, road, and driving behaviors in a real accident. The established model was validated by reproducing the accident in a simulation. The effects of vehicle speed, the height of the cargo’s center of gravity, tractor-semitrailer interactions, and the radius of the circular curve on container semitrailer rollover were investigated using the established model. The results reveal that if the height of the container semitrailer is within the limits in Chinese standards and the container semitrailer is moving at a speed of less than 45.3 km/h, it can safely travel through a loop ramp with a circular curve radius of 60 m. The height of the cargo’s center of gravity and the lateral load transfer ratio have a negative relationship, and a higher center of gravity reduces the safe speed for a container semitrailer traveling through a loop ramp. During rollover, the rear axle of the semitrailer first begins to roll over and then drives the tractor to overturn.

## 1 Introduction

Container semitrailers are extensively used for medium- and long-distance road transportation because they can transport large cargo volumes, are efficient, and are easy to load and unload. However, they also have a large loading mass and high center of gravity; moreover, interactions between tractors and semitrailers often lead to poor lateral stability when driving on curves, and these vehicles are prone to rollover accidents [1–3]. Freeway interchange loop ramps have a small radius and large front- and rear-road alignment changes; thus, they might be unsafe in nonroutine scenarios, and the rate of container semitrailer rollover accidents on these curves is high [4, 5]. Therefore, studying the mechanisms of container semitrailer rollover accidents on freeway loop ramps is critical for improving freeway operation management, safety, and service level [6, 7].

Container semitrailers comprise tractors and semitrailers, which are connected using a traction pin and saddle. In contrast to a single-unit truck, a semitrailer is influenced by the coupling effects of the two vehicle bodies, and its dynamics are more complex than are those of a single-unit truck. When driven on curves, semitrailers not only have similar instabilities to single-unit trucks but also have unique instabilities, which primarily manifest as yaw and roll instabilities [8]. Vehicle, road, and weather factors affect the occurrence of lateral instability in semitrailers. Several scholars have investigated the vehicle factors by establishing vehicle dynamics models by using simulation software [9–13]. Some researchers have also used simulations to investigate the influence of geometric road parameters and the road pavement friction coefficient on the stability of semitrailers moving on curves; these parameters include the radius of horizontal curves, superelevation, and longitudinal gradient [14–16]. Semitrailers are large; thus, some scholars have investigated the effect of crosswind on their lateral stability under severe weather conditions by conducting wind tunnel tests or using the TruckSim simulation software [17–20]. To improve the safety of semitrailers moving on curves, some academics have established models of semitrailer rollovers to predict risk to warn drivers and enable drivers to take timely corrective action by adjusting the vehicle body’s attitude, thereby preventing rollovers [21–25]. In summary, investigations of the mechanisms of container semitrailer rollover accidents while driving on curves are primarily conducted through simulations. Because real-world road tests of semitrailer rollovers are risky, are expensive, and have poor repeatability, few scholars have performed real-world semitrailer tests for verification. In studies that performed such tests [26, 27], to ensure safety, the driving speed of semitrailers did not reach the limit value for rollover. The results of simulation studies without real-world vehicle verification and real-world data have discrepancies.

In response to this challenge, an accident case in which a container semitrailer rolled over on the loop ramp of a trumpet-shaped interchange was investigated in this research. This case was simulated by establishing vehicle, road, and driving behavior models in TruckSim on the basis of data obtained from an in-depth investigation of the accident, such as road geometry parameters and the vehicle driving status, and validating the established models and evaluation indices. We then investigated the influences of vehicle and road factors on the rollover of the container semitrailer and obtained the vehicle’s speed threshold and rollover state for each factor. The findings of this study can be used to provide recommendations for the safe operation of container semitrailers on loop ramps and as references for future research in this area.

## 2 Materials and methods

### 2.1 Background of the accident case

**Fig 1:**
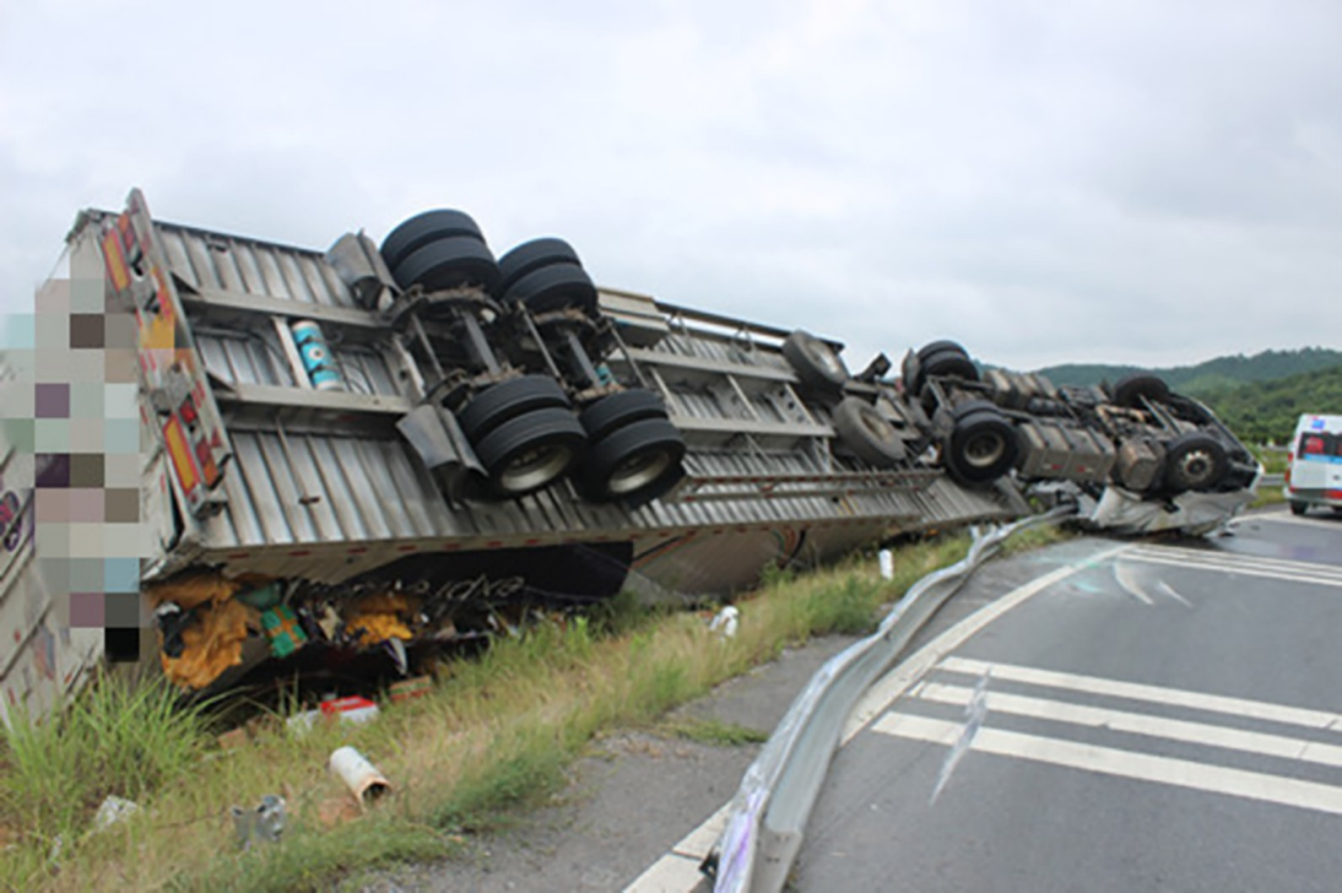
Final resting state of the container semitrailer.

**Fig 2:**
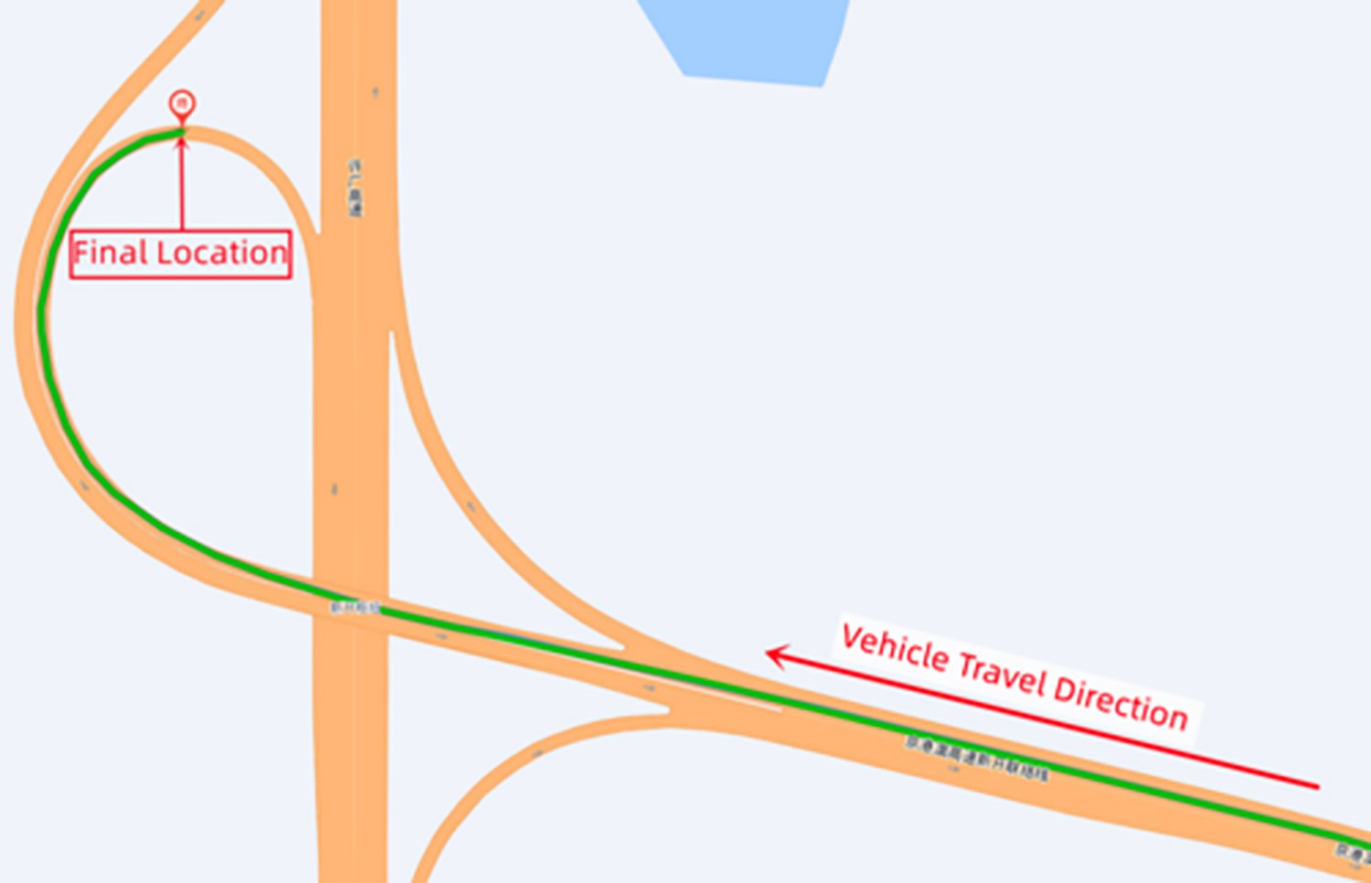
Vehicle trajectory.

On June 18, 2022, a rollover accident occurred while a container semitrailer was traveling along the S18 Xinkai Freeway to the Xinkai interchange loop ramp in Yueyang, Hunan Province, China; this accident resulted in the driver and passenger dying instantly. Fig 1 depicts the accident vehicle’s ultimate stopping state. The accident vehicle was a container semitrailer with a two-axle tractor and two-axle semitrailer (Fig 1). The tractor’s curb weight and outline dimensions were 6800 kg and 6.04 m × 2.495 m × 3.6 m (length, width, and height), respectively. The overall dimensions of the heavy semitrailer were 15.75 m × 3.0 m × 4.45 m (length, width, and height), and the container dimensions were 15.75 m × 3.0 m × 3.45 m (length, width, and height); the curb weights of the semitrailer chassis and container were 3400 and 3650 kg, respectively. The mass of vehicle’s cargo was 17 210 kg. The road section on which the accident occurred is a right-turn loop ramp with a speed limit of 40 km/h, a radius of 60 m, a superelevation of 7%, and a longitudinal slope of −2.45%; the road is made of asphalt and was dry at the time of the accident. Fig 2 illustrates the accident vehicle’s path and its ultimate resting position.

### 2.2 Theoretical mechanical model

**Fig 3:**
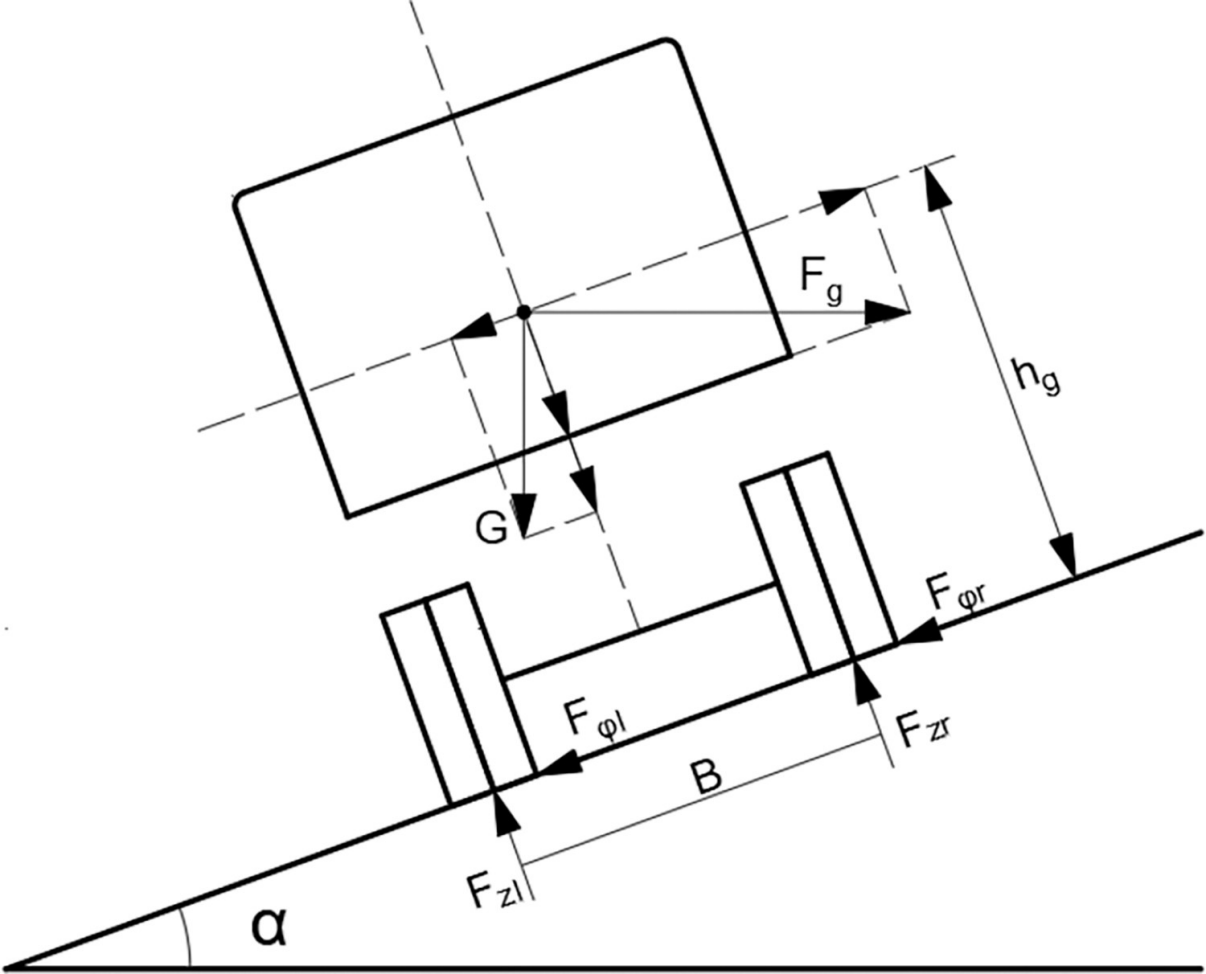
Force diagram of a vehicle on a curve.

When a vehicle is driven at a constant speed on a horizontal straight road, the vertical loads of the left- and right-side tires are identical; the vertical loads on the front and rear axles are not transferred between the tires, and the vehicle is stable. When a vehicle enters a curved section of road, centrifugal forces and lateral tire friction combine to create a rollover moment, which causes the weight on the wheels on the inside of the curve to be transferred to the wheels on the outside of the curve. As the centrifugal force of the vehicle increases, the wheels on the inside of the curve begin to lift off the road, and the vehicle eventually overturns. If the curve has superelevation, the vehicle’s gravity component reduces the centrifugal force, thereby improving the vehicle’s driving stability. Fig 3 illustrates the forces acting on a vehicle moving around a curve under the assumption that the vehicle is a rigid body that is unaffected by tire and suspension deformation.

In Fig 3, *F*_*zl*_ is the support force for the left-side tire, *F*_*zr*_ is the support force for the right-side tire, *F*_*φl*_ is the lateral friction force on the left-side tire, *F*_*φr*_ is the lateral friction force on the right-side tire, *G* is the total mass of the vehicle, *F*_*g*_ is the centrifugal force, *h*_*g*_ is the height of the center of gravity, and *B* is the wheeltrack.

A critical vehicle rollover state occurs if the vertical force exerted by the road on the inner wheel is 0 N; at this time, the moment equation for the contact point between the outer wheel and the road is applicable. This equation is expressed as follows:

$$\frac{GB}{2}\cos\;\alpha +Gh_{g}\;\sin\;\alpha +F_{g}\frac{B}{2}\sin\;\alpha =F_{g}h_{g}\;\cos\;\alpha$$ (1)

Eq (2) is obtained by expanding Eq (1).


$$\frac{mg}{2}B\;\cos\;\alpha +mgh_{g}\;\sin\;\alpha +\frac{m{v_{h}}^{2}}{2r}B\;\sin\;\alpha =\frac{m{v_{h}}^{2}}{r}h_{g}\;\cos\;\alpha$$ (2)

Where *m* is the total mass of the vehicle and *r* is the radius of the road’s horizontal curve.

Typically, the road’s cross-slope angle *α* is small; thus, cos *α* ≈ 1, and sin *α* ≈ tan *α* ≈ *i*_*h*_, where *i*_*h*_ is the superelevation. Eq (3), which is obtained by rearranging Eq (2), is used to determine the critical speed *v*_*h*_ at which rollover occurs for a vehicle traveling on a curve.


$$v_{h}=3.6\sqrt{\frac{B+2h_{g}i_{h}}{2h_{g}-Bi_{h}}gr}$$ (3)

Eq (3) clearly reveals that (assuming that driver error, pavement damage, foreign objects, or other abnormal factors are absent), wheeltrack, the height of the center of gravity, superelevation, and turning radius are the main variables influencing the critical vehicle speed at which rollover occurs.

### 2.3 Construction of the simulation model

Mechanical Simulation Corporation created the vehicle dynamics simulation software known as TruckSim (version 2019.0, Mechanical Simulation Corporation, Ann Arbor, Michigan, USA) specifically for simulating the movements of trucks, buses, and trailers. In this study, vehicle, driver, and road models were established in TruckSim on the basis of the in-depth investigation data obtained from the studied accident.

#### 2.3.1 Vehicle model

In TruckSim, a model of the vehicle’s dynamics was constructed, including the entire vehicle arrangement, driving system, steering system, braking system, transmission system, and other elements. The “2A Cab Over w/2A Trl (Susp.Cab)” model was used in TruckSim as the vehicle model. Similar to the accident vehicle, this model comprises two elements, namely a heavy tractor and heavy semitrailer. Table 1 presents the key indicators of the heavy tractor.

**Table 1:** Main parameters of the heavy tractor.

Parameter	Value	Parameter	Value
Length/mm	6040	Curb weight /kg	6800
Width/mm	2495	Total traction mass/kg	35000
Height/mm	3600	Tire size	295/80R22.5
Wheelbase/mm	3800	Roll inertia around the x-axis/ (kg·m-2)	2283.9
Front wheeltrack/mm	2040	Pitch inertia around the y-axis/ (kg·m-2)	35402.8
Rear wheeltrack/mm	1820	Yaw inertia around the z-axis/ (kg·m-2)	34802.6

A semitrailer model was created in TruckSim by using the “15.5t Trailer, Tandem, Leaf” module; the key characteristics of this semitrailer are listed in Table 2. The semitrailer’s total mass is affected by its load; therefore, the weight was set as the actual cargo weight of the accident vehicle (17 210 kg). The longitudinal position of the cargo’s center of gravity is 8.2 m from the hitch, and its lateral position coincides with the container’s geometric center.

**Table 2:** Main parameters of the heavy semitrailer.

Parameter	Value	Parameter	Value
Length/mm	15750	Curb weight /kg	7050
Width/mm	3000	Tire size	275/70R22.5
Height/mm	3450	Roll inertia around the x-axis/ (kg·m-2)	9959.7
Wheelbase/mm	9545+1310	Pitch inertia around the y-axis/ (kg·m-2)	171336
Wheeltrack/mm	1820	Yaw inertia around the z-axis/ (kg·m-2)	179992

The height of the center of gravity of a container semitrailer is primarily affected by the heights of the centers of gravity of the semitrailer chassis and container (including cargo), as presented in Eq (4) [28]. The height of the center of gravity of the semitrailer chassis is fixed, whereas the container’s center of gravity is affected by the stacking height and mass distribution of the loaded cargo. In this study, the mass of the cargo was assumed to be uniformly distributed, and the cargo stacking height was assumed to be the container’s height.

$$h_{g}=\frac{h_{1}G_{1}+h_{2}G_{2}}{G_{1}+G_{2}}$$ (4)

where *h*_1_ and *h*_*2*_ are the heights of the center of gravity of the semitrailer chassis center and container (including cargo), respectively. Moreover, *G*_1_ and *G*_2_ denote the masses of the semitrailer chassis and container (including cargo), respectively.

#### 2.3.2 Road model

The geometric characteristics of the road and the pavement friction coefficients were input to the TruckSim three-dimensional road module to complete the road model. The “U-Road, 45.72-m (150-ft) Radius” module was selected to create a roadway horizontal alignment comprising the following segments sequentially: a straight line, transition curve, circular curve, transition curve, circular curve, transition curve, and straight line. For the road longitudinal section, the “Road: Reference Line Elevation” module was selected with the sample interpolation and linear extrapolation settings to ensure that the longitudinal section was smoothly aligned. The “Road: Off-Center Elevation Map, S-L Grid” module was selected as the road cross-section setting, and two-dimensional sample bar interpolation and extrapolation methods were adopted to ensure smooth transitions for the road section’s cross-slope. The ramp studied in this paper is the dry asphalt pavement, and the existing research results show [29] that the adhesion coefficient of dry asphalt pavement is mainly affected by the roughness of the pavement and tire, the contact area between the tire and pavement, and the speed of the vehicle traveling, and the range of its value is 0.7–0.8. Therefore, the friction coefficient of the road pavement was selected from the “Road: Friction Map, S-L Grid” module and set to a minimum value of 0.7.

#### 2.3.3 Driving behavior model

The TruckSim driver control model primarily comprises vehicle speed control, shift control, steering control, and braking control. The vehicle was set to be driven at various constant speeds. An automatic shift control model was used to shift gears in response to changes in driving speed. The braking control module was a braking control model available in TruckSim that enables the vehicle to maintain a constant speed on downslopes. The steering control module was a closed-loop control model with the driver’s preview time and lag time set as 1.5 and 0.15 s [30], respectively. The road centerline was used as the target trajectory for the driving vehicle, and the movement of the vehicle was controlled in accordance with the target trajectory.

### 2.4 Rollover condition

The ground support force acting on the wheels on the inside of a curve is a key indicator of a vehicle’s tendency to roll over on the curve. The lateral load transfer ratio (LTR), which is defined as the ratio of the difference between the vertical loads of the left and right wheels of the same axle to the total vertical load, was selected as the evaluation index for determining rollover. This index can accurately reflect the tendency of the vehicle to roll over during turning and driving, as presented in Eq (5). The LTR has values in the range of [0, 1]. The LTR is 0 when the vertical loads of the wheels on both sides are equal. Vehicles begin to roll over if the wheels on the inside of a curve have a vertical load of 0 N; in this case, the LTR is 1, and the vertical weight acting on the wheels on the inside of the curve is entirely transferred to the wheels on the outside of the curve. According to relevant studies [31, 32], vehicles are in a safe state when 0 < LTR ≤ 0.2, a low-rollover-risk state when 0.2 < LTR ≤ 0.6, a greater-rollover-risk state when 0.6 < LTR ≤ 0.8, and a severe-rollover-risk state when 0.8 < LTR ≤ 1.

$$LTR=|\frac{F_{zln}-F_{zrn}}{F_{zln}+F_{zrn}}|$$ (5)

where *n =* 1, 2, 3, 4. Moreover, *F*_*zln*_ and *F*_*zrn*_ are the ground support forces acting on the left and right wheels of the *n*th axle, respectively.

## 3 Simulated reconstruction of the accident case

From the semitrailer chassis and container box size data in the in-depth accident report, the height of the semitrailer cargo’s center of gravity (h_3_) was calculated to be approximately 2.725 m. Assuming that the cargo mass was uniformly distributed, the container semitrailer in the considered accident case was fully loaded with a cargo mass of 17 210 kg. A trumpet-type interchange loop ramp model was developed from field-measured values obtained from the accident location and relevant design data; Table 3 lists the specific parameters.

**Table 3:** Geometric alignment parameters of the road.

Parameter	Radius/m	Length/m	Station/m	Longitudinal slope/%	Cross slope /%
Straight line		50	(0,50)	-2.45	-2
Transition curve		96.8	(50,146.8)	-2.45	6
Circular curve	125	126.122	(146.8,308.032)	-2.45	6
Transition curve		54.315	(308.032,363.469)	-2.45	7
Circular curve	60	73.799	(363.469,437.268)	-2.45	7
Transition curve		106.667	(437.268,543.935)	0.893	2
Straight line		50	(543.935,643.935)	0	-2

**Fig 4:**
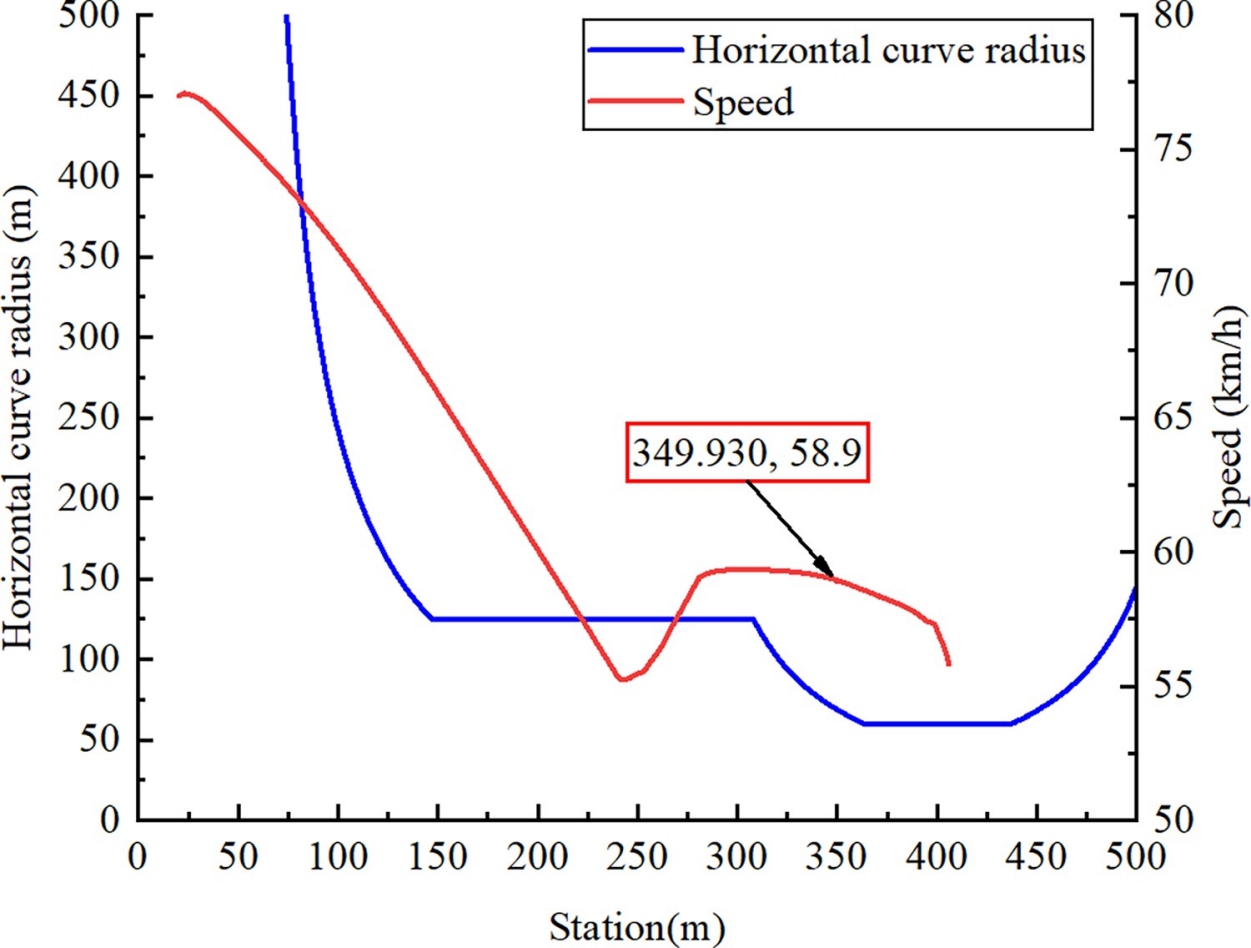
Semitrailer speed and horizontal curve radius.

On the basis of the recorded driving speed and braking conditions from the accident vehicle’s traffic recorder, the container semitrailer entered the loop ramp at a speed of 77 km/h. The driver applied the brakes to reduce its speed to 55 km/h and subsequently released them. The semitrailer accelerated to 58 km/h because of gravity, at which point the driver applied the brakes until the vehicle rolled over. A brake control model based on these braking conditions was created in Simulink such that the speed changes of the simulated container semitrailer were similar to those in the accident case. Fig 4 presents the semitrailer speed and horizontal curve radius at each station (distance from the point at which the vehicle trajectory was start position). At station 349.930 m, the vehicle began to roll over at a speed of 58.9 km/h.

**Fig 5:**
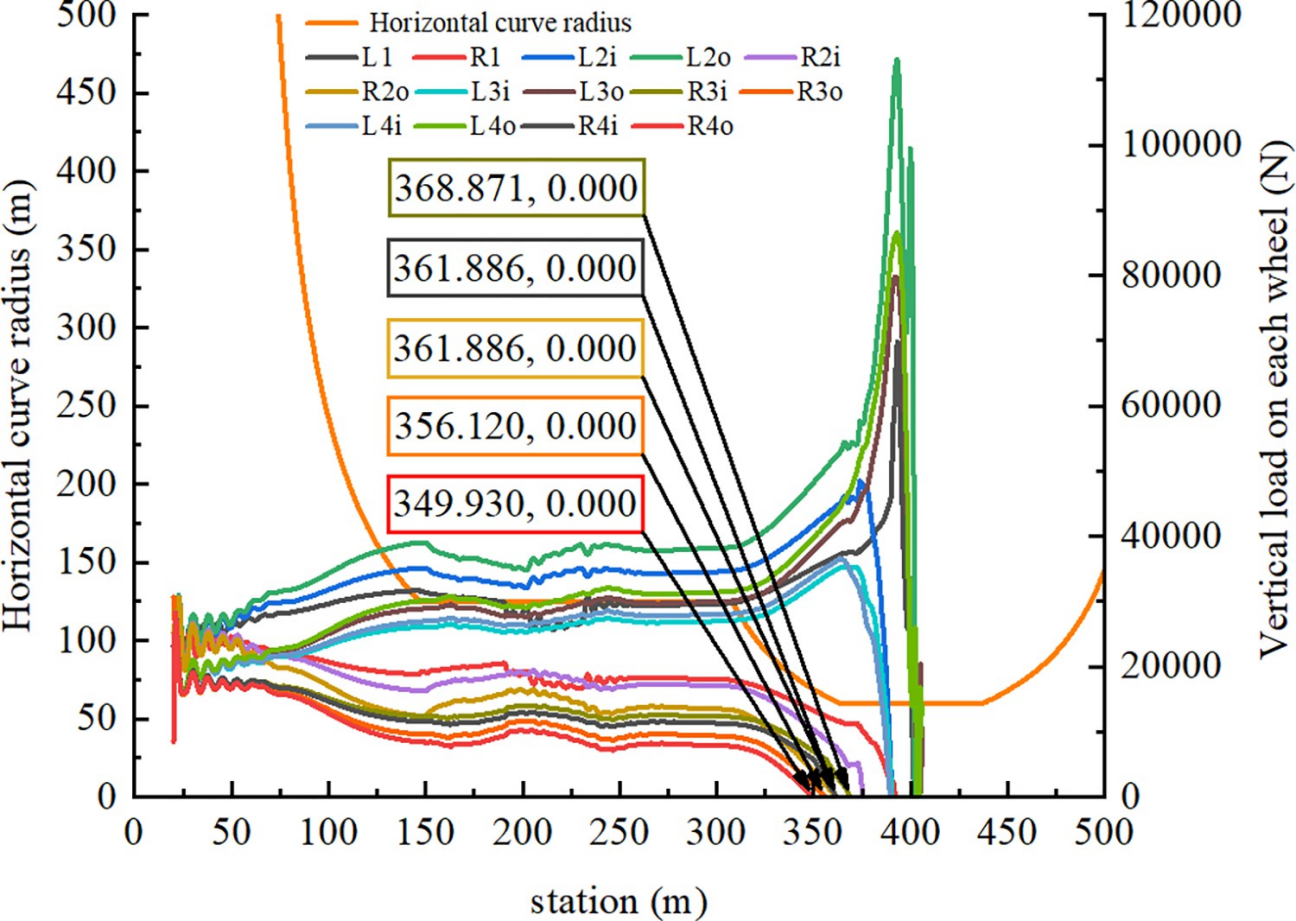
Vertical load curves for every wheel.

**Fig 6:**
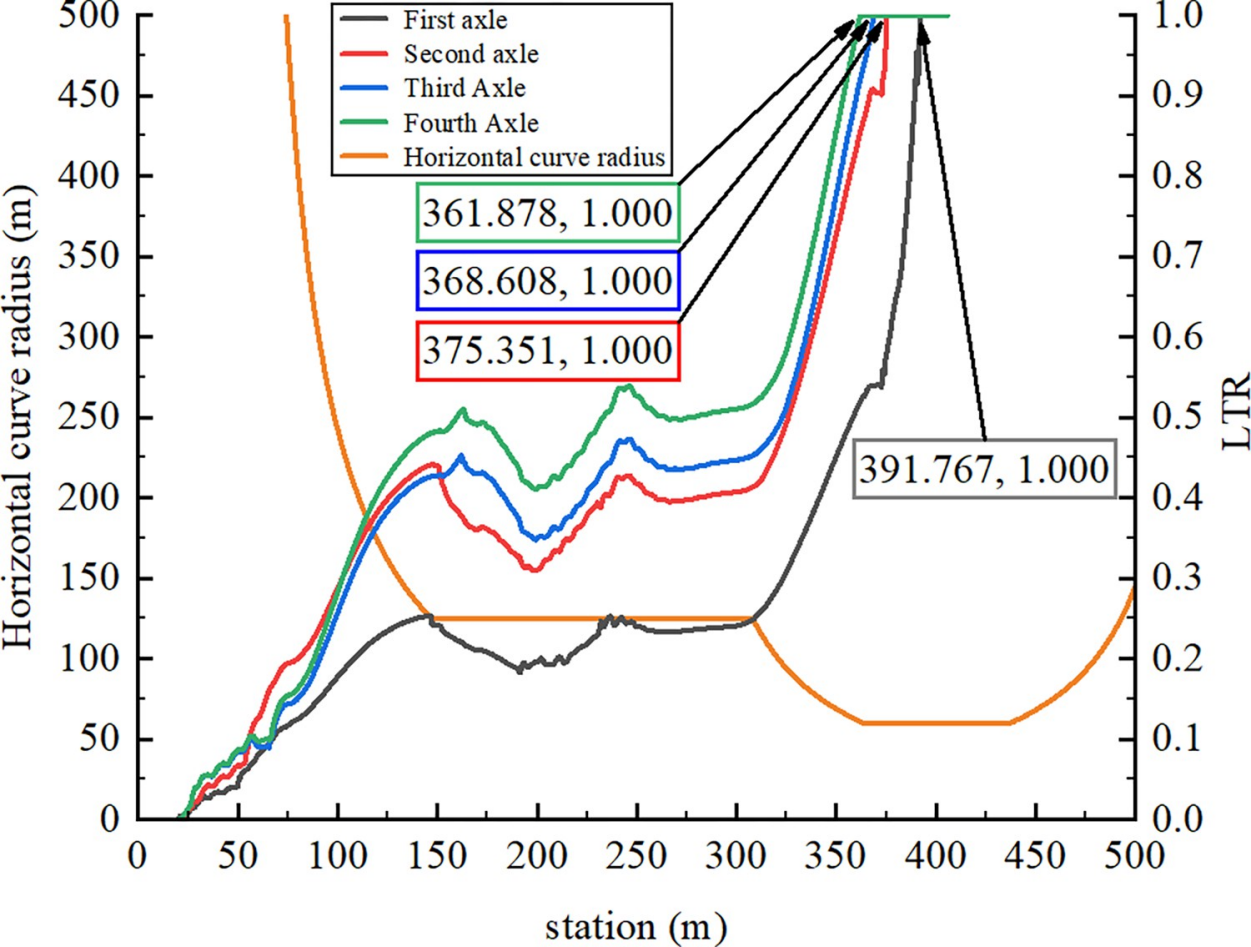
LTR curves for every axle.

**Fig 7:**
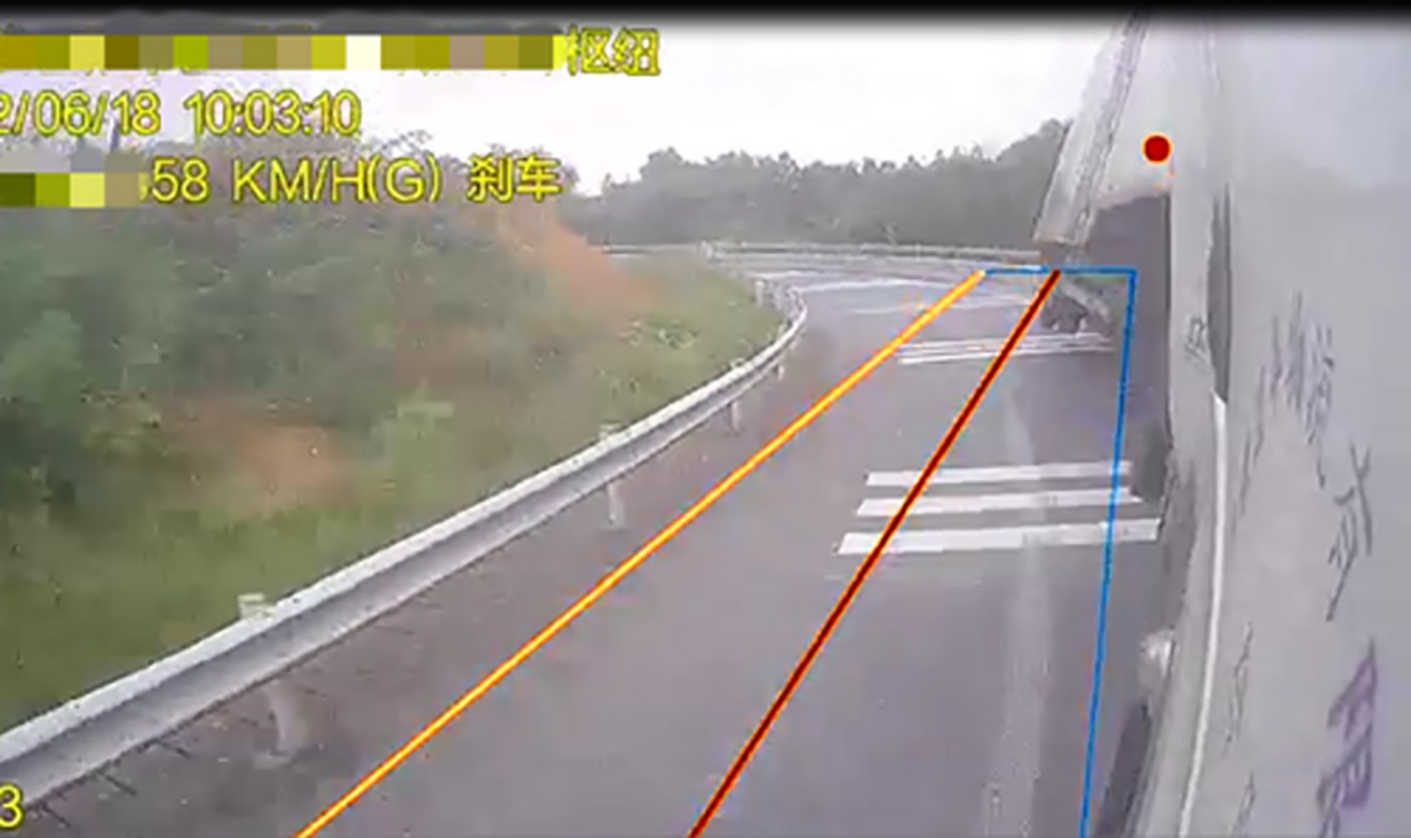
Beginning of the actual container semitrailer rollover.

**Fig 8:**
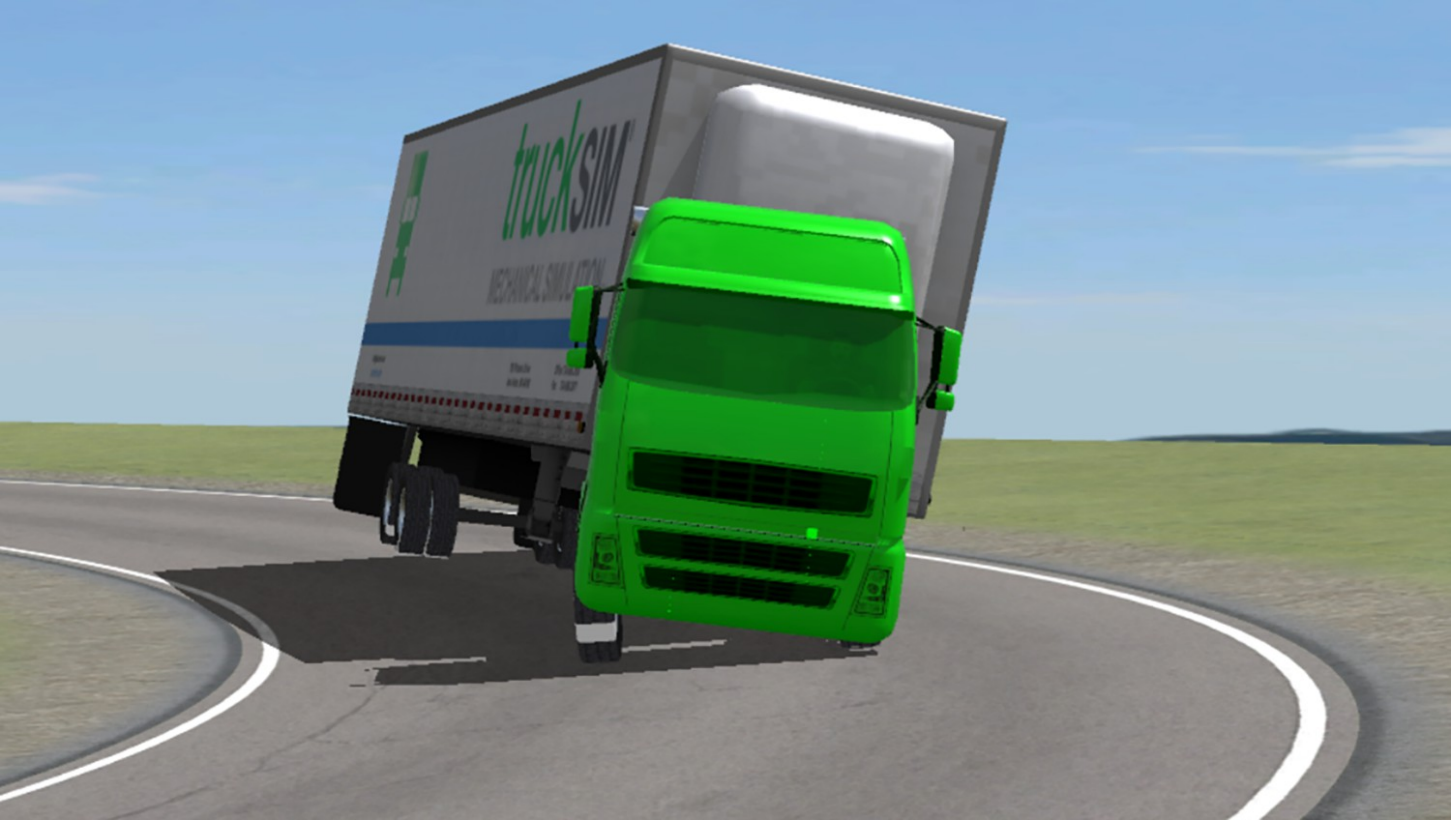
Beginning of the simulated container semitrailer rollover.

As displayed in Fig 5, the vertical force acting on each semitrailer wheel changed during the simulation. Each wheel is denoted with a code in which L or R indicates the left or right side, respectively; a number between 1 and 4 indicates one of the four axles of the tractor and semitrailer; and i or o indicates an inner or outer wheel, respectively. For example, the inner wheel on the right side of the third axle is denoted as R3i. First, at station 349.930 m, the vertical force acting on R4o first reached 0 N. Second, the vertical force on R3o decreased to 0 N at station 356.120 m. Finally, the vertical force between R2o and R4i became 0 N at station 361.886 m. In summary, the right-side wheels of the fourth axle of the semitrailer lifted off the ground first. The tractor’s right wheels on the other axles continued to lift off the ground as the rollover worsened; eventually, the container semitrailer rolled over on a curve with a radius of 60 m. Fig 6 reveals that at station 361.878 m, the LTR of the semitrailer’s fourth axle reached the critical value of 1. These results indicate that LTR can accurately describe semitrailer rollovers. When the vertical force of R4o reached 0 N in the simulation, the semitrailer’s speed was 58.9 km/h. During the rollover, the recorder captured a maximal speed of 59 km/h. On the basis of Eq (3), the critical speed for a semitrailer to overturn is 59.1 km/h. Therefore, the critical rollover speed obtained from the simulation was similar to the theoretical and actual rollover speeds, which indicates that the simulation model is valid. In addition, Fig 5 reveals that when the container semitrailer was driven on the loop ramp, the vertical load on the left-side wheels of each axle increased sharply as the radius of the horizontal curve gradually decreased, whereas the vertical load on the right-side wheels of each axle decreased rapidly to 0 N. The vertical load on the right-side wheels of the container semitrailer’s fourth axle reached 0 N before entering a 60-m circular curve section, which indicated that the semitrailer had begun to roll over in the transition curve section (Figs 7 and 8), and the simulated rollover process was almost identical to the video captured by the accident vehicle’s traffic recorder.

Fig 7 displays the attitude of the accident vehicle body early in the overturning process. The accident vehicle’s overturning process is consistent with the visualization of the simulation results in Fig 8; in both cases, the semitrailer overturned first, followed by the tractor. Moreover, the simulated rollover behavior is the expected behavior for the actual change in the vertical force of each wheel.

**Fig 9:**
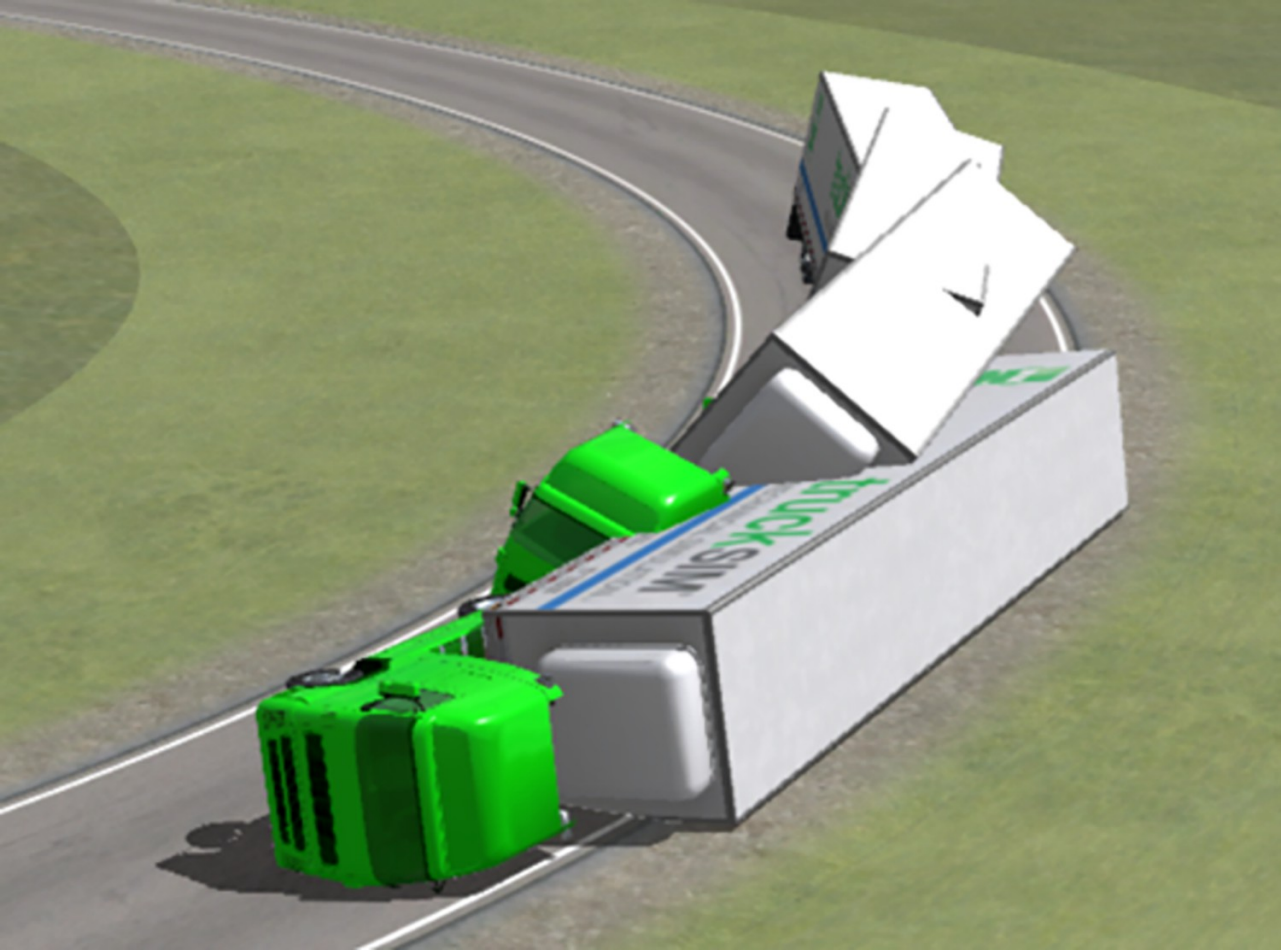
Final state of the simulated container semitrailer rollover.

**Fig 10:**
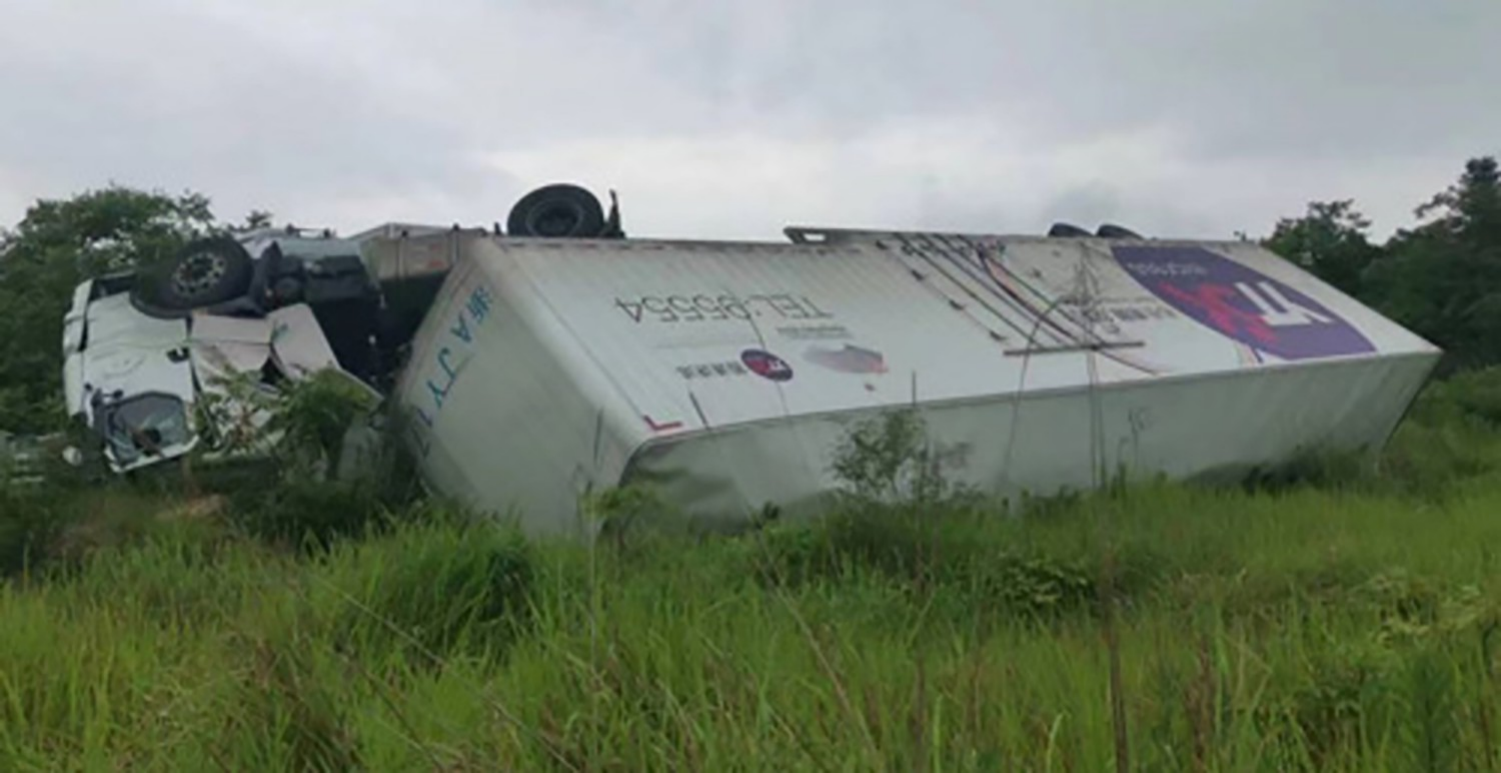
Final state of the actual overturned container semitrailer.

Fig 9 presents an image of the simulated semitrailer rollover. A comparison of Figs 9 and 10 reveals that in both the simulation and actual accident, the left side of the vehicle body was in contact with the ground in the final resting position of the vehicle.

**Fig 11:**
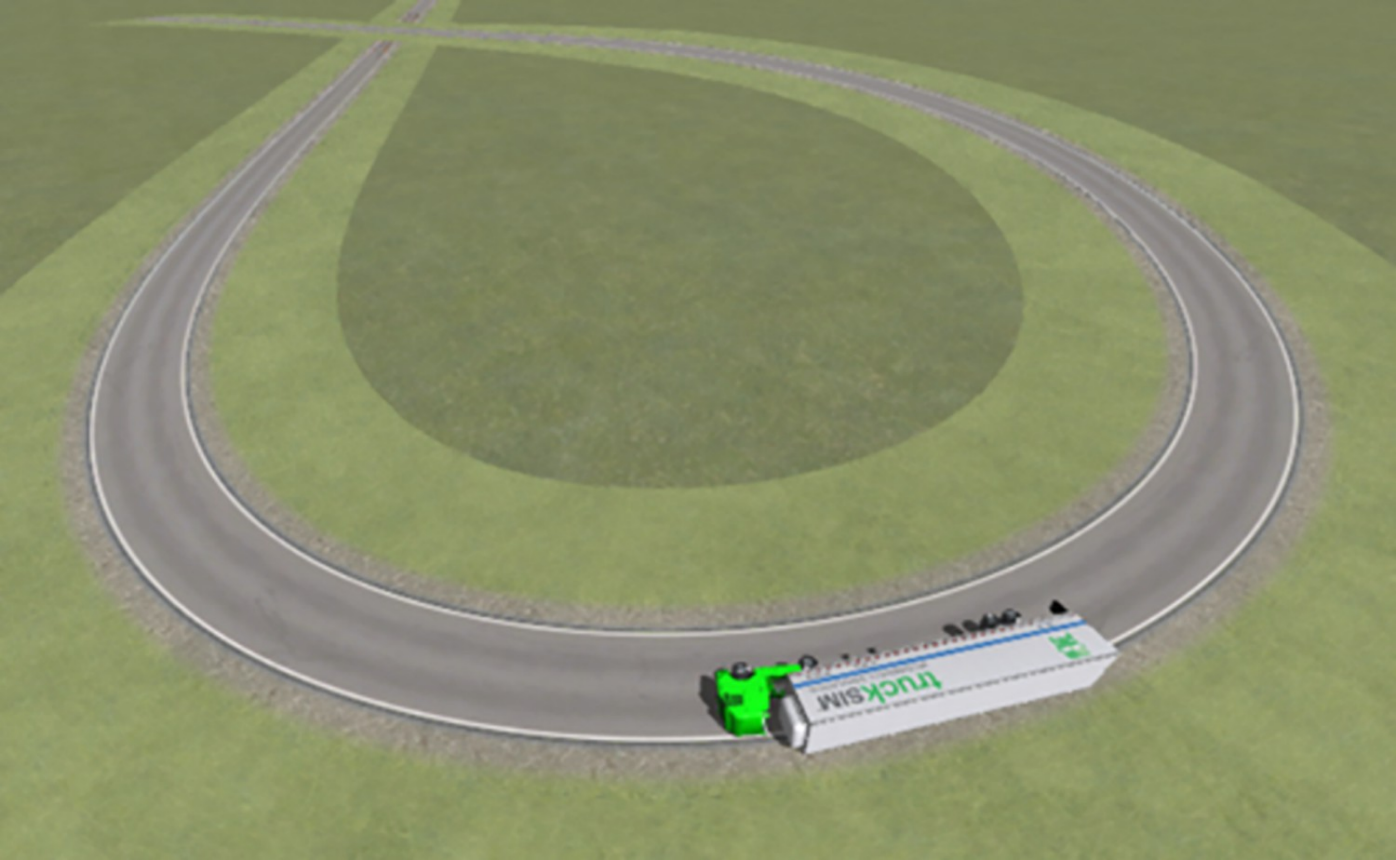
Final rollover positions of the container semitrailer. (Simulation).

**Fig 12:**
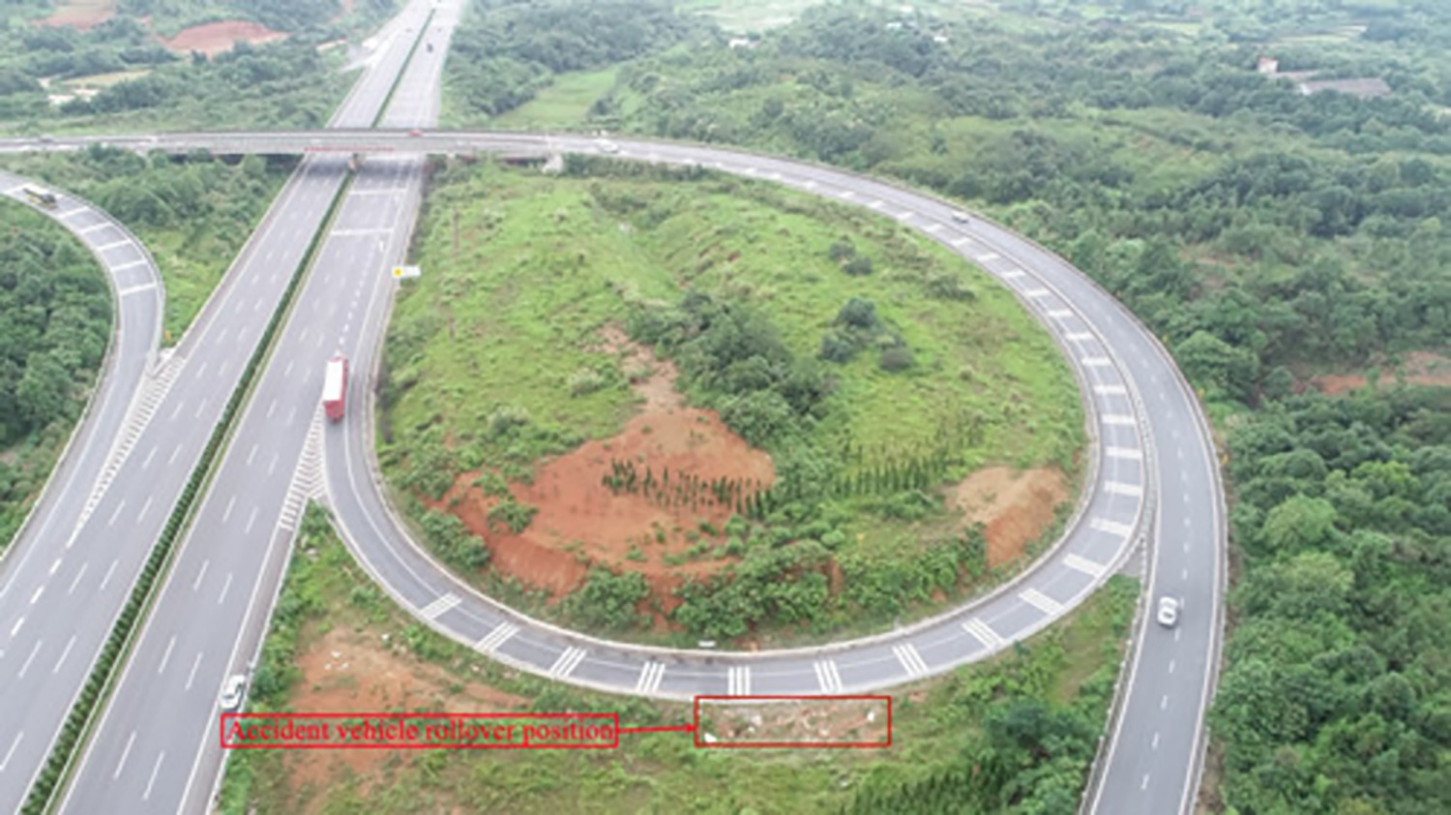
Final rollover positions of the container semitrailer. (Accident case).

Figs 11 and 12 present top views of the final resting positions of the container semitrailer after overturning on the loop ramp in the simulated accident and actual accident, respectively; these positions are consistent. Moreover, on-site data collected at each station were compared with the corresponding simulated data; the difference between the stations of the final simulated and actual stopping positions of the overturned container semitrailer was less than 10 m, and all the corresponding radii of the circular curves were 60 m, which indicated that the simulation accurately reproduced the accident.

## 4 Results and discussion

The theoretical model of rollover mechanics, simulated rollovers for the actual accident, relevant parameters of the accident vehicle, and relevant parameters in Chinese standards were analyzed; these aspects indicate that the primary factors influencing the rollover risk for container semitrailers moving on curves include travel speed, the height of the cargo’s center of gravity, interactions between the tractor and the semitrailer, and the radius of the ramp curve. The produced TruckSim model was used for further investigating how these factors affect the risk of rollover for container semitrailers moving on loop ramps.

### 4.1 Travel speed

To analyze the effect of travel speed, simulations were performed with the road model established in Section 2.3.2. To ensure that other factors do not affect the rollover risk, the load model in the aforementioned simulations was a standard box model in which the load’s center of gravity coincided with the container’s geometric center at a height of 2.75 m, and the load weight was set as 17 210kg. The speed limit of the road section in which the accident occurred is 40 km/h; thus, speeds of 30, 35, 40, 45, and 50 km/h were selected for the simulation tests. The maximum LTR of the container semitrailer in the aforementioned section at each speed was determined through simulation (Table 4).

**Table 4:** LTR values for turning at different speeds.

Test number	Speed(km·h^-1^)	LTR
1	30	0.225
2	35	0.330
3	40	0.467
4	45	0.624
5	50	0.796

**Fig 13:**
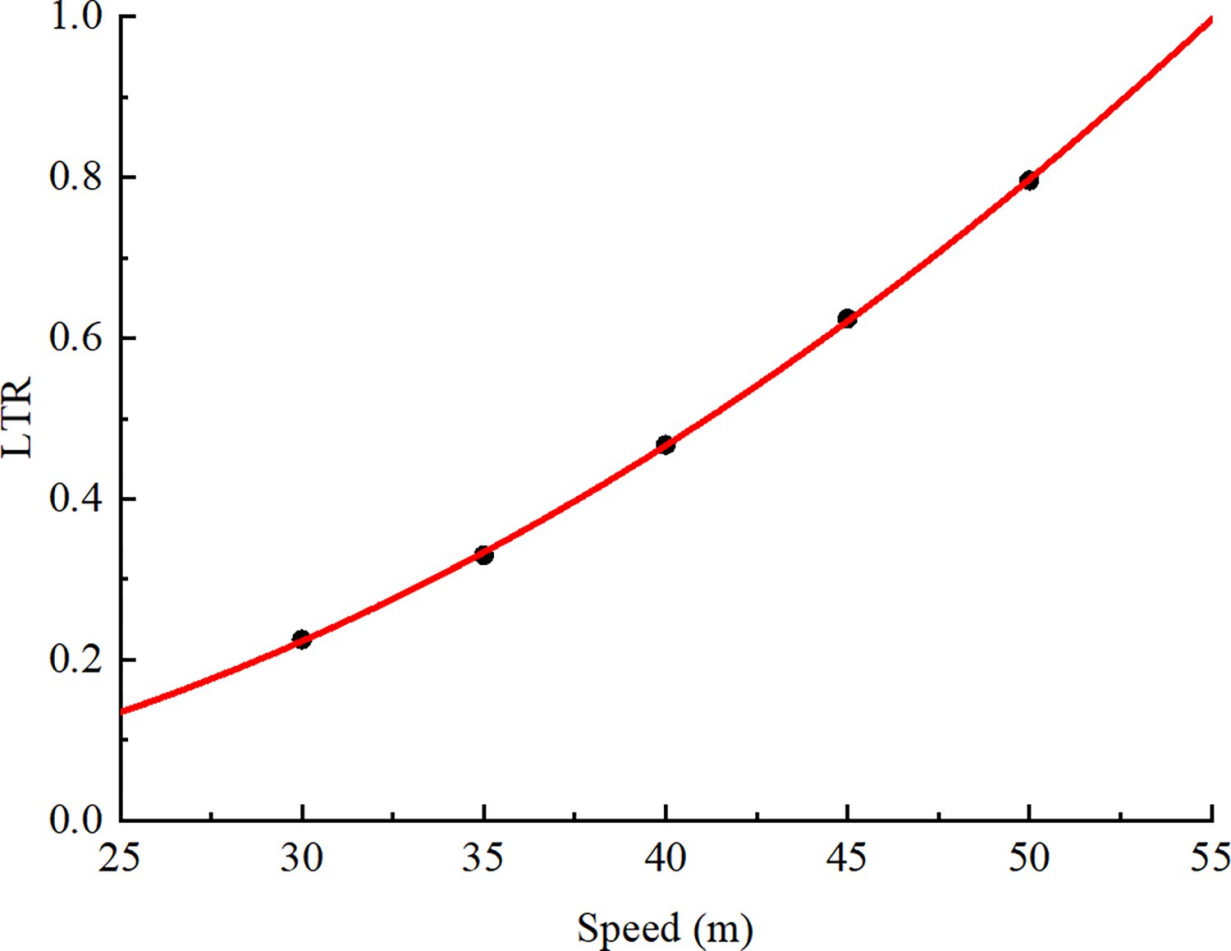
Plot of speed versus LTR (*r* = 60 m).


Fig 13 depicts a plot and best-fit line of the simulation results for the speed and LTR. Eq (6), which was obtained through quadratic regression, represents the relationship between vehicle speed and LTR.

$$LTR=0.0216-0.00648v+0.00044v^{2}$$ (6)

where *v* is the travel speed of the container semitrailer.

For Eq (6), *R*^2^ = 0.992, which indicates a perfect fit. Therefore, Fig 13 reveals that vehicle speed and LTR are significantly and positively correlated, with the correlation coefficient being 0.996 and the significance level being 0.01.

Table 5 suggests that the container semitrailer could successfully traverse the accident section by maintaining a speed below 43.5 km/h. If its speed reached 54.9 km/h, the LTR of its fourth axle reached the rollover limit value of 1, at which point the semitrailer lost stability and rolled over. After the semitrailer began to roll over, the vehicle continued to roll over even if the steering angle was reduced rapidly by turning the steering wheel to increase the semitrailer’s steering radius. In the accident case, the container semitrailer traveled at a maximum speed of 59 km/h while passing through the loop ramp during the rollover. Therefore, the vehicle was traveling at a speed considerably greater than the safe speed of 43.5 km/h for the aforementioned curve and greater than the critical rollover speed of 54.9 km/h. The generated lateral centrifugal force for a container semitrailer travelling at this critical speed is sufficient to cause the wheels on the inside of the curve to detach from the ground, which in turn causes the entire vehicle to roll over. As the container semitrailer passes through the transition curve and enters a road section with a radius smaller than 60 m, the lateral centrifugal force generated by the container semitrailer increases as the radius of the horizontal curve gradually decreases; therefore, the vehicle begins to roll over as it approaches the road section with a curve radius of 60 m. When the container semitrailer enters the circular curve section with the smallest radius from the transition curve section, the container semitrailer overturns completely.

**Table 5:** Speed values corresponding to LTR risk thresholds.

LTR	Speed(km/h)	
	Simulation	Eq (6) calculation
0.6	43.5	44.4
0.8	49.3	50.1
1	54.9	55.1

### 4.2 Influence of the height of the cargo’s center of gravity

The established road model was used for simulations to investigate the effect of the height of the cargo’s center of gravity on the lateral stability of a semitrailer traveling on a curve. The simulated test road had a curve with a radius of 60 m. A vehicle’s speed on a curve is positively correlated with its lateral stability. In the tests conducted using the aforementioned model, the semitrailer’s speed was set as 40 km/h (the speed limit of the road section) to control this effect. The Chinese standard “GB 1589–2016: Limits of Dimensions, Axle Load and Masses for Motor Vehicles, Trailers and Combination Vehicle” states that the maximum outer height of container semitrailers should be 4 m [33]. Assuming that the cargo mass in the container was uniformly distributed and that the cargo’s stack height and container height were the same, the height of the container floor for the accident vehicle was 1 m from the ground. The height of the cargo’s center of gravity is the height of the container floor above the ground plus half the container height. For container heights of 2.4, 2.7, 3.0, 3.3, and 3.6 m, the heights of the cargo’s center of gravity were 2.2, 2.35, 2.5, 2.65, and 2.8 m, respectively. Simulations were conducted to obtain the maximum values of the container semitrailer’s LTR for different heights of the cargo’s center of gravity when the semitrailer passed through a curve at a speed of 40 km/h (Table 6).

**Table 6:** Maximum LTR values for various heights of the cargo’s center of gravity.

Test number	Container height(m)	Cargo gravity center height(m)	LTR
1	2.4	2.200	0.382
2	2.7	2.350	0.405
3	3.0	2.500	0.429
4	3.3	2.650	0.454
5	3.6	2.800	0.481

**Fig 14:**
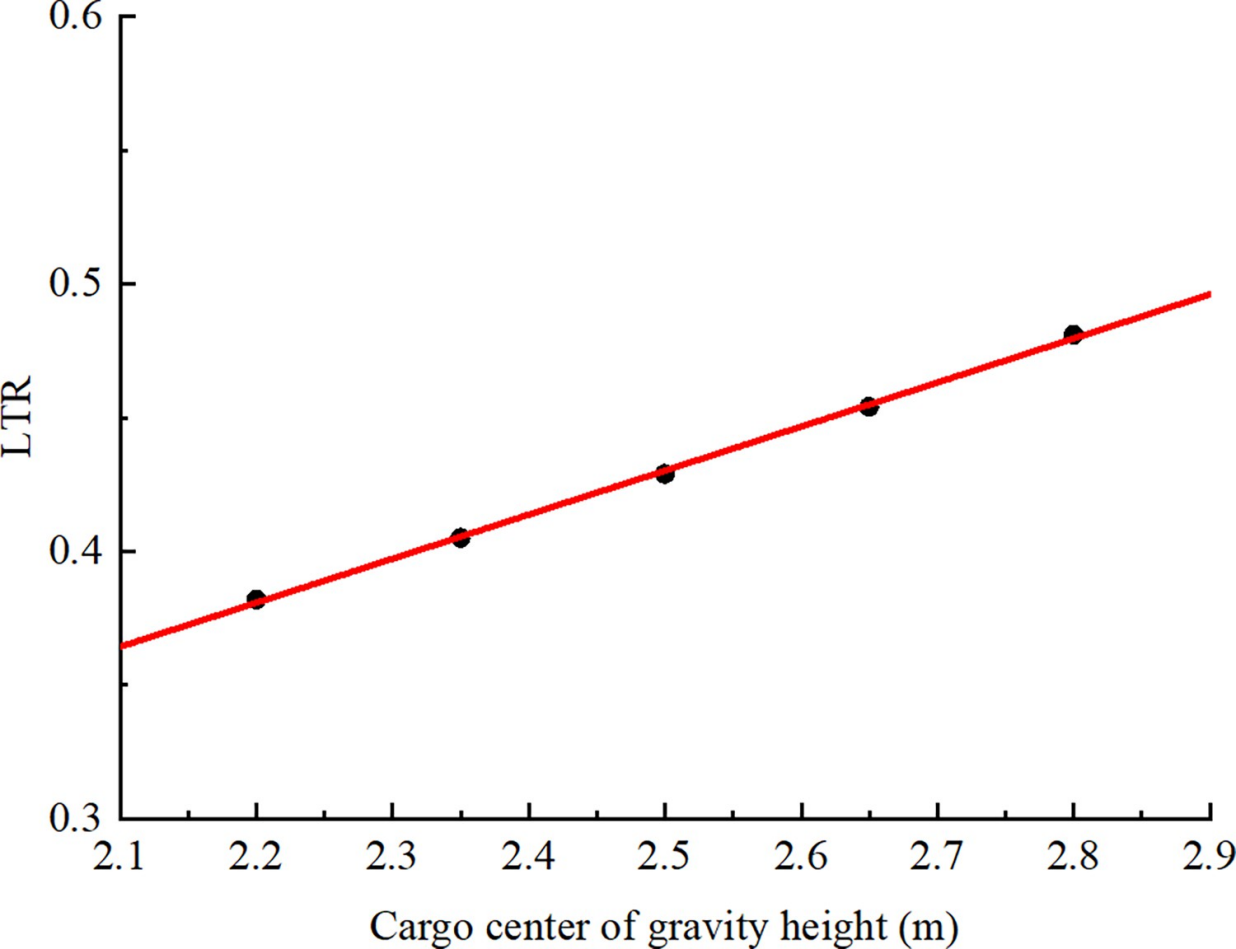
Relationship between the height of the cargo’s center of gravity and LTR (*v* = 40 km/h, *r* = 60 m).


Fig 14 displays a scatter diagram of the simulated results presented in Table 6. Linear regression was performed to determine the relationship between LTR and the height of the cargo’s center of gravity; this result is expressed in Eq (7).

LTR=0.019+0.165h (7)

where *h* is the height of the cargo’s center of gravity.

For the model expressed in Eq (7), the *R*^2^ was 0.999, which indicated a favorable fit. Correlation analysis of the best-fit line (Fig 14) revealed that the correlation coefficient between the LTR and the height of the cargo’s center of gravity for the turning container semitrailer was 1.00 at a significance level of 0.01, which indicated that a significant and positive correlation between these factors. In summary, for a container semitrailer traveling at a constant speed, an increase in the height of the cargo’s center of gravity causes the LTR to increase marginally.

If the total height of a container semitrailer is 4 m, Table 6 reveals that the container height is 3 m and that the height of the cargo’s center of gravity is 0.225 m less than that of the accident vehicle. The maximum LTR for a standards-compliant container semitrailer with a 2.5-m center of gravity that is traveling through a curve with a radius of 60 m at 40 km/h is 0.429. The standards-compliant container semitrailer must be traveling at 45.3 or 57.2 km/h (the critical speeds) to reach the LTR thresholds of 0.6 and 1, respectively. The accident vehicle was initially traveling at 77 km/h. A simulation was performed for a standards-compliant container semitrailer initially traveling at this speed. The simulation revealed that the LTR of both semitrailer axles reached 1, and the wheels on the inside of the curve left the ground. However, the LTR of the tractor axles had not yet reached this critical rollover value. Continuous braking then reduced the vehicle speed, and the vehicle passed through the accident section’s curve without rolling over. However, the actual accident vehicle was excessively tall; its container height was 3.45 m, which corresponds to a height of 2.725 m for the cargo’s center of gravity. In the simulation of this vehicle, the maximum LTR through the curve at a speed of 40 km/h was 0.467, and the LTR was 0.6 at a speed of 43.5 km/h. Moreover, the critical speed of the vehicle (LTR = 1) was 54.9 km/h. A comparison of the aforementioned cases revealed that under the same curve radius, the safe speed for the vehicle with a height of 2.5 m for the cargo’s center of gravity was 4.14% greater than that for the accident vehicle with a height of 2.725 m for the cargo’s center of gravity. This result indicates that ensuring that the height of a container does not exceed the maximum value in the relevant standards and avoiding the high stacking of heavy objects in the container to achieve a low cargo center of gravity can increase the safe speed of a semitrailer through a bend. When the horizontal curve radius is 60 m, Table 7 displays the speed thresholds for the rollover of container semitrailers under different cargo center gravity heights. The theoretical rollover speed thresholds are determined by Eq (3).

**Table 7:** Rollover speed thresholds. (r = 60 m).

Test number	Cargo gravity center height(m)	Rollover speed threshold (km/h)	
		theoretical value	simulation value
1	2.2	64.2	63.0
2	2.35	62.5	61.3
3	2.5	60.4	59.7
4	2.65	58.7	58.0
5	2.8	57.3	56.3

### 4.3 Tractor–semitrailer interactions

**Fig 15:**
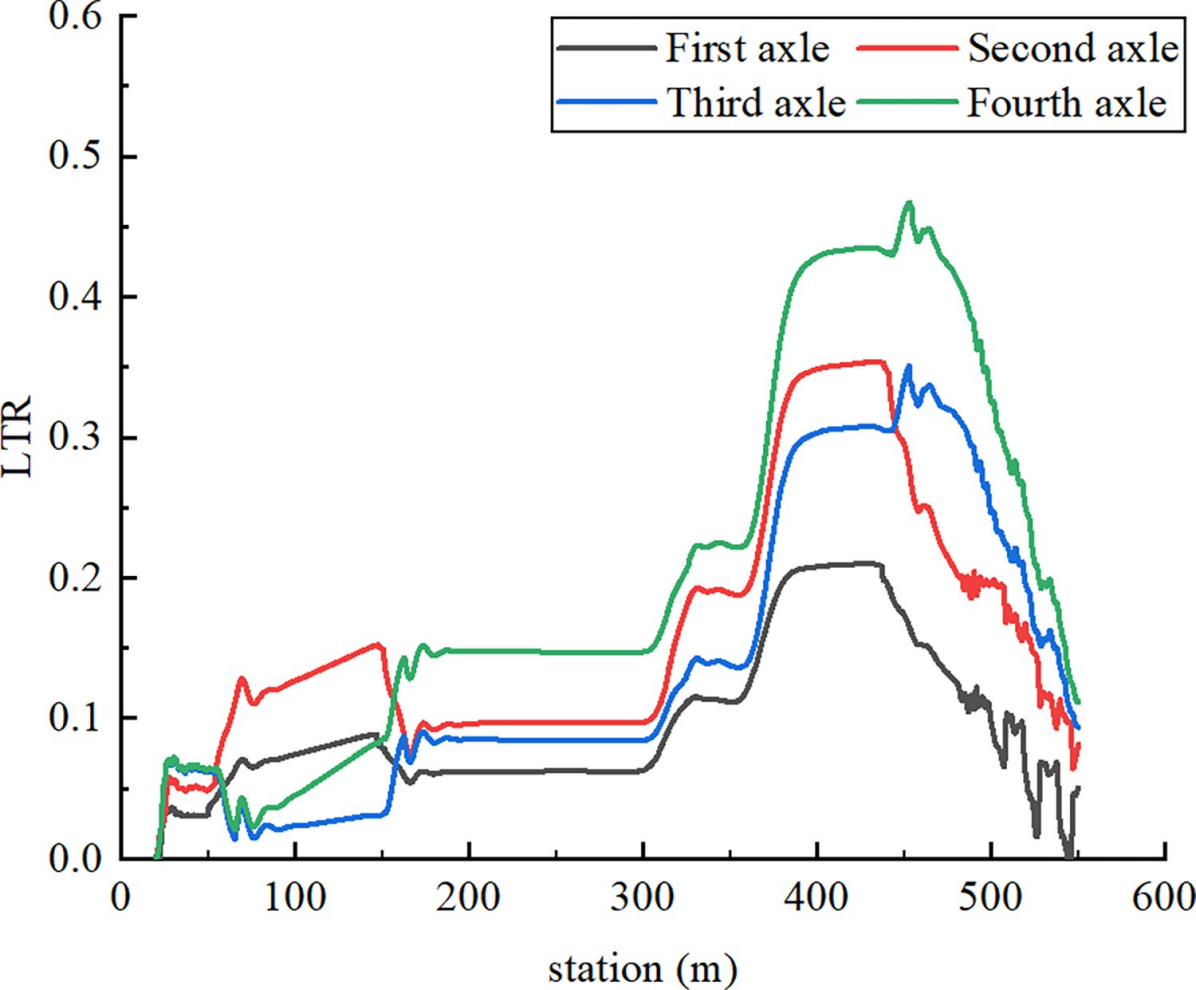
LTR of each axle (*v* = 40 km/h, *r* = 60 m).

Semitrailers and tractors are hitched together through a saddle; each of these parts has freedom of lateral yaw and roll at the hitch such that the roll movement of the tractor during turning and driving is transferred to the semitrailer through the saddle. In addition, the large distance between the saddle and the rear axle of a semitrailer as well as the vehicle body’s flexibility can amplify the roll effects at the rear of the semitrailer. When a tractor and semitrailer are subjected to the same lateral acceleration but with the aforementioned rear amplification, the semitrailer can easily roll excessively, thereby resulting in the inner wheels of the trailer axle set leaving the ground because of an excessive transfer of lateral load. This phenomenon causes the inner wheels of the tractor drive axle and steering axle to leave the ground, and the entire vehicle eventually rolls over. To obtain the LTR for each axle of the semitrailer (Fig 15), the semitrailer model established in Section 2.3.1 was again applied in simulation experiments with a vehicle with a cargo center of gravity at 2.75 m and a cargo mass of 17 210 kg that passed through the road model (Section 2.3.2) at 40 km/h. During turning, the fourth axle exhibited the highest LTR among the axles, whereas the first axle exhibited the smallest LTR. Therefore, during turning, the lateral load transfer to the rear axle was most likely to be excessive, thereby resulting in the load on one side being completely transmitted to the other side and the wheels lifting off the ground followed by rollover.

**Fig 16:**
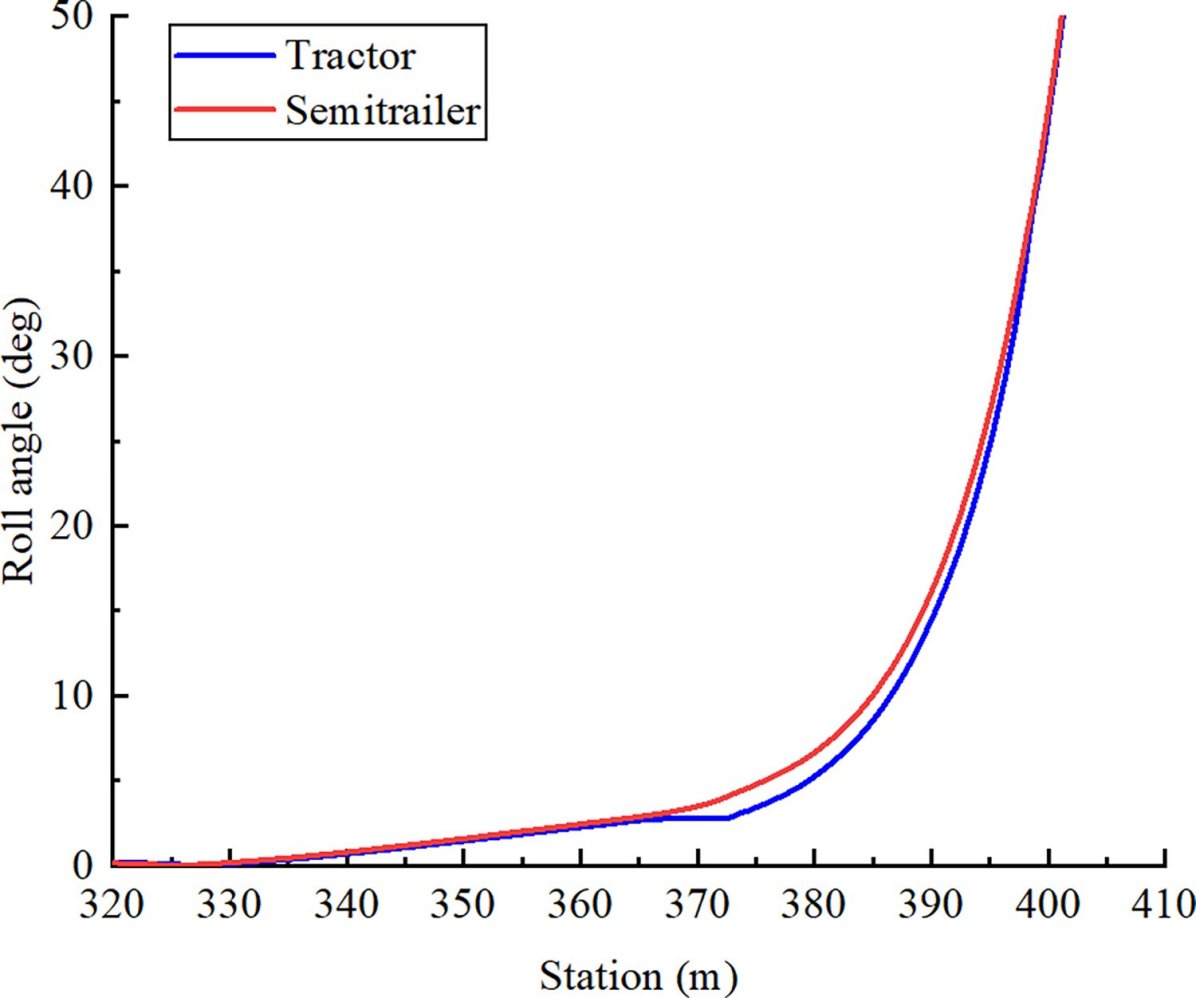
Curves of the roll angles of the tractor and semitrailer.

**Fig 17:**
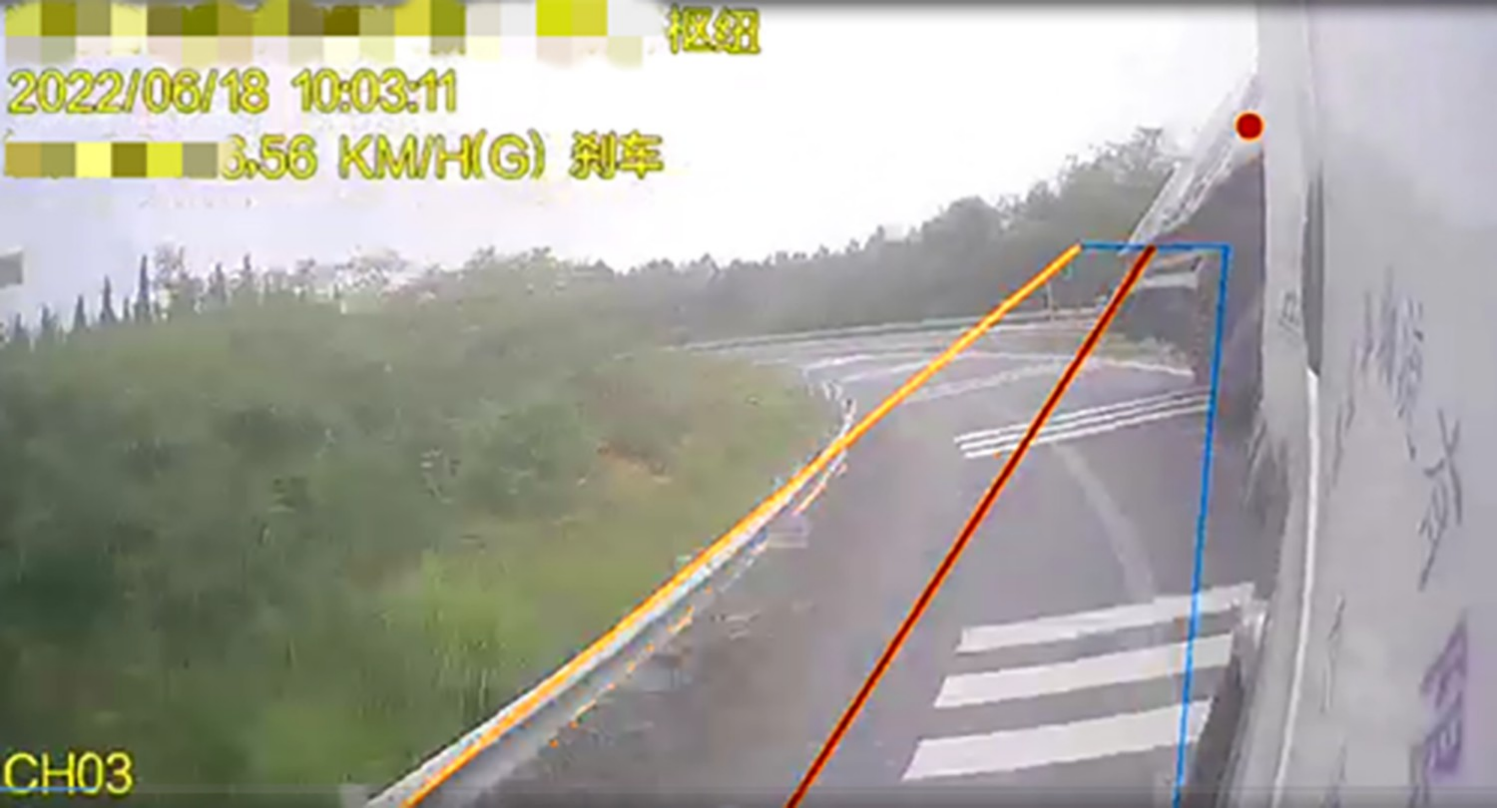
Image of the vehicle rollover during the accident.

Fig 16 was obtained through the simulated reconstruction of the accident case. This figure shows the curves of the changes in the roll angles of the tractor and semitrailer. Before entering the segment with the minimum curve radius, the roll angle values of the tractor and semitrailer were similar; as the radius of the horizontal curve decreased, the roll angle values of the tractor and semitrailer increased. However, during rollover, the semitrailer’s roll angle increased considerably before the tractor’s roll angle; thus, the semitrailer tended to roll over first and subsequently caused the tractor to roll over. At a given station, the roll angle of the tractor was less than that of the semitrailer during rollover. The roll angle of the tractor was transmitted to the semitrailer through the saddle and then amplified in the semitrailer, thereby further increasing the roll angle of the semitrailer. Data from the accident vehicle’s recorder indicates that when the container semitrailer rolled over, the semitrailer’s roll angle was greater than the tractor’s roll angle (Fig 17). Therefore, in the accident, the semitrailer overturned first, and the tractor overturned as the roll angle of the semitrailer increased. A comparison of the accident case and simulation results revealed that both rollover processes were nearly identical; both processes clearly demonstrated that the tractor’s rollover angle was amplified in the semitrailer after being transmitted through the saddle.

### 4.4 Effect of horizontal curve radius

To investigate the effect of bend radius on the rollover of a container moving at 40 km/h, simulations were performed for circular curves with radii of 60, 70, 80, 90, and 100 m. These curves had a design speed of 40 km/h and a superelevation of 7% in accordance with the standard “Design Specification for Alignment (JTG D20-2017)” [34]. The simulated vehicle moved through each bend at 40 km/h. The height of the cargo’s center of gravity and its weight were set to those of the accident vehicle. The maximum LTR for each bend radius was obtained from the simulation (Table 8).

**Table 8:** LTR values for different curve radii.

Test number	Curve radius (m)	LTR
1	60	0.467
2	70	0.381
3	80	0.319
4	90	0.275
5	100	0.238

**Fig 18:**
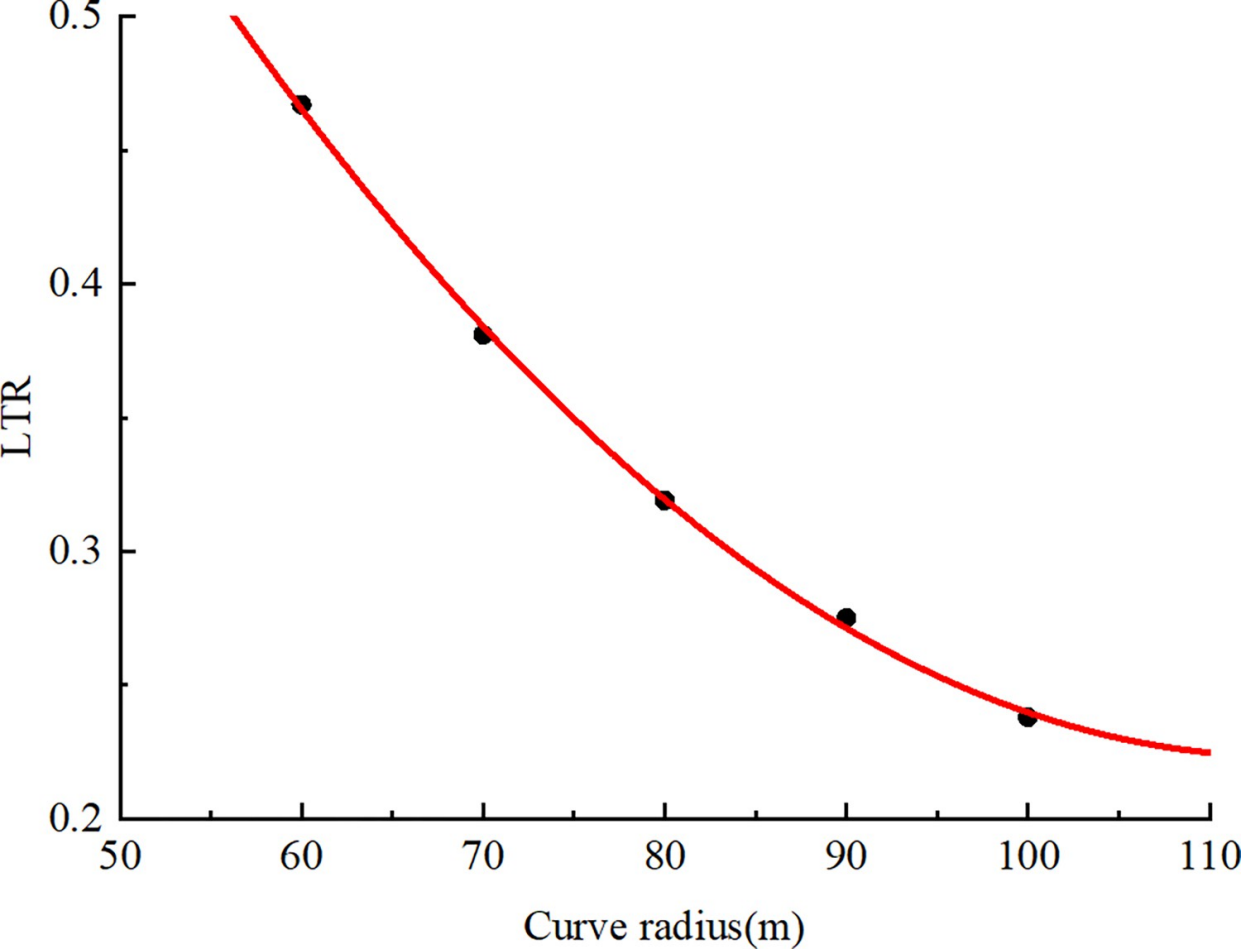
Relationship between curve radius and LTR (*v* = 40 km/h).

The results presented in Table 8 were plotted as a scatter plot (Fig 18), and curvilinear regression was performed to obtain the best-fit line, which is expressed as follows:

$$LTR=1.3009142857-0.0188971429r+0.0000828571r^{2}$$ (8)

For Eq (8), *R*^2^ = 0.999, which indicates a favorable fit. The correlation coefficient between the LTR and the bend radius was −0.985 at a significance level of 0.01; therefore, the LTR and bend radius were significantly and negatively correlated with each other. In the accident case, the loop ramp’s minimum circular curve radius was 60 m, which is the minimum circular curve radius for the design speed of 40 km/h. The critical speed is the minimum speed for a container semitrailer to overturn on a curve with a radius of 60 m. In the accident case, the excessive height of the vehicle resulted in a greater stacked cargo height and therefore higher center of gravity; the results presented in Section 4.2 reveal that this phenomenon decreased the critical rollover speed. If the overall height of the container semitrailer is 4 m (the maximum height limit of the relevant standard) and the container height is 3 m, driving through the circular curve section at a speed less than or equal to the design speed is safe because the critical speed is 57.2 km/h. However, the accident vehicle’s speed before the accident reached 59 km/h, and the container was excessively tall, which caused the vehicle to begin to roll over in the transition curve before entering the 60-m-radius segment of the circular curve. The simulation results indicate that as the radius of the circular curve increases, the critical rollover speed also increases. Therefore, in road design, extreme bend radii should be avoided; if the minimum bend radius must be used, stepped speed limit signs should be posted to ensure that vehicle speeds are substantially lower than the critical rollover speed. Table 9 shows the rollover speed thresholds of container semitrailers under different horizontal curve radii when the cargo center gravity height of the semitrailer is 2.725 m, where the theoretical rollover speed thresholds are obtained from Eq (3).

**Table 9:** Rollover speed thresholds. (h_3_ = 2.725 m).

Test number	horizontal curve radius (m)	Rollover speed threshold (km/h)	
		theoretical value	simulation value
1	2.2	57.7	55.9
2	2.35	62.4	59.9
3	2.5	66.7	64.0
4	2.65	70.8	67.8
5	2.8	74.6	72.9

## 5 Conclusions

The main conclusions of this study are as follows:

- When a container semitrailer is traveling on a curve, speed significantly affects rollover risk. The speed limit for interchange loop ramps (such as in the accident case) where freeways connect with other freeways should be reduced in steps, and drivers should be reminded of the speed limit by providing speed measurements, reminders to drive cautiously, and warnings regarding the sharp curve such that container semitrailers can reduce their speed to a safe cornering speed before entering the curve section with the minimum radius.
- Taller container semitrailers have a higher center of gravity, which reduces the speed at which they can safely pass through a loop ramp and therefore significantly increases the risk of rollover when traveling at excessive speed. To decrease or completely prevent the risk of rollover for container semitrailers on circular ramps, relevant departments should increase their supervision of container heights.
- Container semitrailers are affected by interactions between the tractor and the semitrailer, and the increase in the height of the entire vehicle’s center of gravity when the container semitrailer is loaded causes the tractor’s roll to be amplified on the semitrailer during turning. When container semitrailer rollover occurs, the semitrailer first rolls over and then causes the tractor to roll over. To avoid rollover for turning container semitrailers, the change in the LTR of each axis of the semitrailer should be considered, and the vehicle’s steering angle should be adjusted, or the driving speed should be reduced in a timely manner.
- The minimum bend radius for a circular ramp affects the safe speed of a container semitrailer; therefore, the extreme bend radius in the specification should be avoided if possible in road design. If the minimum bend radius is used, speed limits and sharp curves should be clearly marked.

## Supporting information

S1 Dataset (XLSX)

S2 Dataset (XLSX)

S3 Dataset (XLSX)

S4 Dataset (XLSX)

S5 Dataset (XLSX)
